# (Methoxy­carbon­yl)hydrazinium chloride monohydrate

**DOI:** 10.1107/S1600536808031474

**Published:** 2008-10-04

**Authors:** Jian-Wu Xie, Lu-Ping Lv, Wen-Bo Yu, Wei-Wei Li, Xian-Chao Hu

**Affiliations:** a Department of Chemical Engineering, Hangzhou Vocational and Technical College, Hangzhou 310018, People’s Republic of China; b Research Center of Analysis and Measurement, Zhejiang University of Technology, Hangzhou 310014, People’s Republic of China

## Abstract

In the title compound, C_2_H_7_N_2_O_2_^+^·Cl^−^·H_2_O, the non-H atoms of the cation are approximately coplanar. The organic cations, chloride ions and water mol­ecules are linked into a two-dimensional network parallel to the *bc* plane by N—H⋯O, N—H⋯Cl and O—H⋯Cl hydrogen bonds.

## Related literature

For applications of benzaldehyde­hydrazone derivatives, see: Parashar *et al.* (1988 ▶); Hadjoudis *et al.* (1987 ▶). For the crystal structure of a nickel methyl­carbazate complex, see: Song *et al.* (2003 ▶).
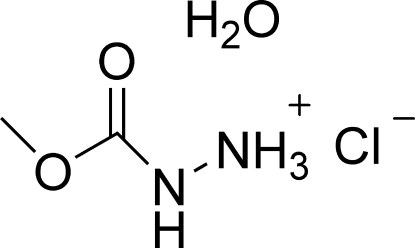


## Experimental

### 

#### Crystal data

C_2_H_7_N_2_O_2_^+^·Cl^−^·H_2_O
*M* *_r_* = 144.56
Monoclinic, 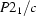

*a* = 12.6621 (13) Å
*b* = 7.6444 (7) Å
*c* = 6.6948 (7) Å
β = 97.199 (4)°
*V* = 642.91 (11) Å^3^
*Z* = 4
Mo *K*α radiation
μ = 0.53 mm^−1^
*T* = 123 (2) K
0.28 × 0.24 × 0.23 mm

#### Data collection

Bruker SMART CCD area-detector diffractometer
Absorption correction: multi-scan (*SADABS*; Bruker, 2002 ▶) *T*_min_ = 0.861, *T*_max_ = 0.881
7105 measured reflections
1445 independent reflections
1360 reflections with *I* > 2σ(*I*)
*R*_int_ = 0.021

#### Refinement

*R*[*F* ^2^ > 2σ(*F* ^2^)] = 0.030
*wR*(*F* ^2^) = 0.080
*S* = 1.04
1445 reflections
97 parameters
3 restraints
H atoms treated by a mixture of independent and constrained refinement
Δρ_max_ = 0.59 e Å^−3^
Δρ_min_ = −0.30 e Å^−3^

### 

Data collection: *SMART* (Bruker, 2002 ▶); cell refinement: *SAINT* (Bruker, 2002 ▶); data reduction: *SAINT*; program(s) used to solve structure: *SHELXS97* (Sheldrick, 2008 ▶); program(s) used to refine structure: *SHELXL97* (Sheldrick, 2008 ▶); molecular graphics: *SHELXTL* (Sheldrick, 2008 ▶); software used to prepare material for publication: *SHELXTL*.

## Supplementary Material

Crystal structure: contains datablocks I, global. DOI: 10.1107/S1600536808031474/ci2680sup1.cif

Structure factors: contains datablocks I. DOI: 10.1107/S1600536808031474/ci2680Isup2.hkl

Additional supplementary materials:  crystallographic information; 3D view; checkCIF report

## Figures and Tables

**Table 1:** Hydrogen-bond geometry (Å, °)

*D*—H⋯*A*	*D*—H	H⋯*A*	*D*⋯*A*	*D*—H⋯*A*
N1—H1*A*⋯O1*W*	0.92 (2)	1.84 (2)	2.743 (2)	167 (2)
N1—H1*B*⋯Cl1^i^	0.93 (2)	2.20 (2)	3.1152 (14)	168 (2)
N1—H1*C*⋯O1^ii^	0.89 (2)	2.00 (2)	2.8443 (17)	158 (2)
O1*W*—H1*W*⋯Cl1^iii^	0.85 (2)	2.41 (3)	3.2172 (16)	161 (3)
N2—H2⋯Cl1^iv^	0.86 (1)	2.33 (1)	3.1833 (13)	171 (2)
O1*W*—H2*W*⋯Cl1	0.82 (2)	2.58 (3)	3.1959 (14)	133 (3)

Symmetry codes: (i) 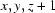; (ii) 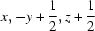; (iii) 
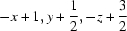
; (iv) 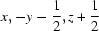.

**Fig. 1.**
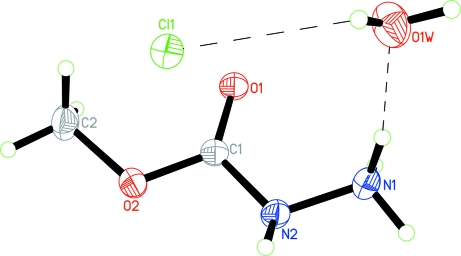
The molecular structure of the title compound, showing 30% probability displacement ellipsoids and the atomic numbering. Hydrogen bonds are shown as dashed lines.

**Fig. 2.**
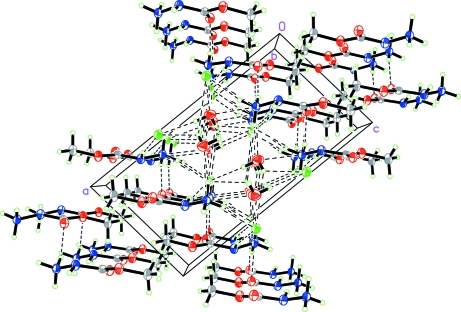
The crystal packing of the title compound, viewed approximately along the c axis. Hydrogen bonds are shown as dashed lines.

## supplementary crystallographic information

### Comment

Benzaldehydehydrazone derivatives have received considerable attention for a long time due to their pharmacological activity (Parashar *et al.*, 1988) and their photochromic properties (Hadjoudis *et al.*, 1987). The title compound is an important intermediate in the synthesis of benzaldehydehydrazone derivatives. We report here the crystal structure of the title compound (Fig. 1).

In the cation, atoms O1, O2, N2, C1 and C2 are coplanar (r.m.s. deviation 0.029 Å) and atom N1 deviates by 0.260 (2) Å from the C1/C2/O1/O2/N2 plane. The bond lengths and angles in the organic cation are comparable to those in a related structure (Song *et al.*, 2003).

The molecules are linked into a two-dimensional network parallel to the *bc* plane by N–H···O, N—H···Cl and O—H···Cl hydrogen bonds involving the water molecule and chloride ions (Table 1 and Fig.2).

### Experimental

Methyl hydrazinecarboxylate (0.90 g, 0.01 mol) was dissolved in ethanol- dilute HCl and single crystals suitable for X-ray analysis were obtained by slow evaporation at room temperature (m.p. 463–465 K).

### Refinement

O- and N-bound H atoms were located in a difference map and were refined with O-H and N2-H2 distances restrained to 0.85 (2) Å and 0.87 (1) Å, respectively. The methyl H atoms were disordered over two orientations and their occupancies were initially refined and later fixed at 0.75 and 0.25, with C-H = 0.96 å and *U*_iso_(H) = 1.5*U*_eq_(C).

### Crystal data

**Table:**

C_2_H_7_N_2_O_2_^+^·Cl^−^·H_2_O	*F*(000) = 304
*M**_r_* = 144.56	*D*_x_ = 1.494 Mg m^−^^3^
Monoclinic, *P*2_1_/*c*	Mo *K*α radiation, λ = 0.71073 Å
Hall symbol: -P 2ybc	Cell parameters from 1122 reflections
*a* = 12.6621 (13) Å	θ = 1.6–25.0°
*b* = 7.6444 (7) Å	µ = 0.53 mm^−^^1^
*c* = 6.6948 (7) Å	*T* = 123 K
β = 97.199 (4)°	Block, colourless
*V* = 642.91 (11) Å^3^	0.28 × 0.24 × 0.23 mm
*Z* = 4	

### Data collection

**Table:**

Bruker SMART CCD area-detector diffractometer	1445 independent reflections
Radiation source: fine-focus sealed tube	1360 reflections with *I* > 2σ(*I*)
graphite	*R*_int_ = 0.021
φ and ω scans	θ_max_ = 27.5°, θ_min_ = 1.6°
Absorption correction: multi-scan (SADABS; Bruker, 2002)	*h* = −14→15
*T*_min_ = 0.861, *T*_max_ = 0.881	*k* = −9→9
7105 measured reflections	*l* = −8→8

### Refinement

**Table:**

Refinement on *F*^2^	Primary atom site location: structure-invariant direct methods
Least-squares matrix: full	Secondary atom site location: difference Fourier map
*R*[*F*^2^ > 2σ(*F*^2^)] = 0.030	Hydrogen site location: inferred from neighbouring sites
*wR*(*F*^2^) = 0.080	H atoms treated by a mixture of independent and constrained refinement
*S* = 1.05	*w* = 1/[σ^2^(*F*_o_^2^) + (0.0389*P*)^2^ + 0.2916*P*] where *P* = (*F*_o_^2^ + 2*F*_c_^2^)/3
1445 reflections	(Δ/σ)_max_ = 0.001
97 parameters	Δρ_max_ = 0.59 e Å^−^^3^
3 restraints	Δρ_min_ = −0.30 e Å^−^^3^

### Special details

**Table:**

Geometry. All e.s.d.'s (except the e.s.d. in the dihedral angle between two l.s. planes) are estimated using the full covariance matrix. The cell e.s.d.'s are taken into account individually in the estimation of e.s.d.'s in distances, angles and torsion angles; correlations between e.s.d.'s in cell parameters are only used when they are defined by crystal symmetry. An approximate (isotropic) treatment of cell e.s.d.'s is used for estimating e.s.d.'s involving l.s. planes.
Refinement. Refinement of *F*^2^ against ALL reflections. The weighted *R*-factor *wR* and goodness of fit *S* are based on *F*^2^, conventional *R*-factors *R* are based on *F*, with *F* set to zero for negative *F*^2^. The threshold expression of *F*^2^ > σ(*F*^2^) is used only for calculating *R*-factors(gt) *etc*. and is not relevant to the choice of reflections for refinement. *R*-factors based on *F*^2^ are statistically about twice as large as those based on *F*, and *R*- factors based on ALL data will be even larger.

### Fractional atomic coordinates and isotropic or equivalent isotropic displacement parameters (Å^2^)

**Table:**

	*x*	*y*	*z*	*U*_iso_*/*U*_eq_	Occ. (<1)
O1	0.83051 (8)	0.17986 (13)	0.89504 (15)	0.0351 (2)	
O2	0.88232 (9)	−0.10131 (13)	0.86201 (17)	0.0380 (3)	
N1	0.69905 (11)	0.07910 (18)	1.1544 (2)	0.0343 (3)	
H1A	0.6428 (16)	0.110 (3)	1.061 (3)	0.047 (6)*	
H1B	0.6731 (14)	0.031 (3)	1.267 (3)	0.042 (5)*	
H1C	0.7342 (16)	0.174 (3)	1.203 (3)	0.047 (5)*	
N2	0.77164 (10)	−0.04049 (16)	1.08177 (18)	0.0337 (3)	
H2	0.7430 (14)	−0.1422 (16)	1.064 (3)	0.045 (5)*	
C1	0.82736 (11)	0.02547 (18)	0.93676 (19)	0.0287 (3)	
C2	0.93930 (14)	−0.0505 (2)	0.6966 (3)	0.0441 (4)	
H2A	0.9769	−0.1498	0.6530	0.066*	0.75
H2B	0.9892	0.0405	0.7402	0.066*	0.75
H2C	0.8896	−0.0086	0.5869	0.066*	0.75
H2D	0.9269	0.0712	0.6670	0.066*	0.25
H2E	0.9146	−0.1191	0.5799	0.066*	0.25
H2F	1.0141	−0.0700	0.7332	0.066*	0.25
O1W	0.55461 (12)	0.1831 (2)	0.8367 (2)	0.0561 (4)	
H1W	0.4948 (17)	0.216 (4)	0.867 (4)	0.095 (10)*	
H2W	0.537 (3)	0.109 (4)	0.751 (4)	0.115 (12)*	
Cl1	0.64630 (3)	−0.09761 (5)	0.54932 (5)	0.03710 (14)	

### Atomic displacement parameters (Å^2^)

**Table:**

	*U*^11^	*U*^22^	*U*^33^	*U*^12^	*U*^13^	*U*^23^
O1	0.0401 (6)	0.0279 (5)	0.0388 (5)	−0.0011 (4)	0.0106 (4)	0.0032 (4)
O2	0.0416 (6)	0.0320 (5)	0.0426 (6)	0.0058 (4)	0.0136 (5)	0.0020 (4)
N1	0.0351 (7)	0.0369 (7)	0.0323 (6)	−0.0037 (5)	0.0101 (5)	−0.0023 (5)
N2	0.0381 (7)	0.0275 (6)	0.0371 (6)	−0.0032 (5)	0.0113 (5)	0.0010 (5)
C1	0.0276 (6)	0.0293 (6)	0.0285 (6)	−0.0009 (5)	0.0013 (5)	0.0005 (5)
C2	0.0430 (9)	0.0471 (9)	0.0455 (8)	0.0020 (7)	0.0182 (7)	−0.0039 (7)
O1W	0.0544 (8)	0.0635 (9)	0.0482 (7)	0.0110 (7)	−0.0022 (6)	−0.0172 (6)
Cl1	0.0391 (2)	0.0390 (2)	0.0344 (2)	0.00628 (14)	0.00935 (14)	0.00276 (13)

### Geometric parameters (Å, °)

**Table:**

O1—C1	1.2146 (17)	C2—H2A	0.96
O2—C1	1.3272 (17)	C2—H2B	0.96
O2—C2	1.4486 (19)	C2—H2C	0.96
N1—N2	1.4243 (17)	C2—H2D	0.96
N1—H1A	0.92 (2)	C2—H2E	0.96
N1—H1B	0.93 (2)	C2—H2F	0.96
N1—H1C	0.89 (2)	O1W—H1W	0.847 (17)
N2—C1	1.3661 (17)	O1W—H2W	0.819 (18)
N2—H2	0.860 (9)		
			
C1—O2—C2	115.31 (12)	H2B—C2—H2C	109.5
N2—N1—H1A	114.1 (12)	O2—C2—H2D	109.5
N2—N1—H1B	109.4 (12)	H2A—C2—H2D	141.1
H1A—N1—H1B	109.2 (16)	H2B—C2—H2D	56.3
N2—N1—H1C	109.5 (13)	H2C—C2—H2D	56.3
H1A—N1—H1C	110.5 (18)	O2—C2—H2E	109.5
H1B—N1—H1C	103.5 (17)	H2A—C2—H2E	56.3
C1—N2—N1	114.74 (12)	H2B—C2—H2E	141.1
C1—N2—H2	118.7 (13)	H2C—C2—H2E	56.3
N1—N2—H2	110.5 (13)	H2D—C2—H2E	109.5
O1—C1—O2	126.08 (13)	O2—C2—H2F	109.5
O1—C1—N2	123.84 (13)	H2A—C2—H2F	56.3
O2—C1—N2	109.90 (12)	H2B—C2—H2F	56.3
O2—C2—H2A	109.5	H2C—C2—H2F	141.1
O2—C2—H2B	109.5	H2D—C2—H2F	109.5
H2A—C2—H2B	109.5	H2E—C2—H2F	109.5
O2—C2—H2C	109.5	H1W—O1W—H2W	102 (3)
H2A—C2—H2C	109.5		
			
C2—O2—C1—O1	−8.8 (2)	N1—N2—C1—O1	12.3 (2)
C2—O2—C1—N2	175.92 (13)	N1—N2—C1—O2	−172.27 (12)

### Hydrogen-bond geometry (Å, °)

**Table:**

*D*—H···*A*	*D*—H	H···*A*	*D*···*A*	*D*—H···*A*
N1—H1A···O1W	0.92 (2)	1.84 (2)	2.743 (2)	167 (2)
N1—H1B···Cl1^i^	0.93 (2)	2.20 (2)	3.1152 (14)	168 (2)
N1—H1C···O1^ii^	0.89 (2)	2.00 (2)	2.8443 (17)	158 (2)
O1W—H1W···Cl1^iii^	0.85 (2)	2.41 (3)	3.2172 (16)	161 (3)
N2—H2···Cl1^iv^	0.86 (1)	2.33 (1)	3.1833 (13)	171 (2)
O1W—H2W···Cl1	0.82 (2)	2.58 (3)	3.1959 (14)	133 (3)

Symmetry codes: (i) *x*, *y*, *z*+1; (ii) *x*, −*y*+1/2, *z*+1/2; (iii) −*x*+1, *y*+1/2, −*z*+3/2; (iv) *x*, −*y*−1/2, *z*+1/2.
